# Maxillary Sinus Augmentation with Decellularized Bovine Compact Particles: A Radiological, Clinical, and Histologic Report of 4 Cases

**DOI:** 10.1155/2017/2594670

**Published:** 2017-03-02

**Authors:** Antonio Scarano

**Affiliations:** Department of Medical, Oral and Biotechnological Sciences and CeSi-MeT, University of Chieti-Pescara, Via dei Vestini 31, 66100 Chieti, Italy

## Abstract

*Background*. One of the most problematic regions for endosseous implants is the posterior maxilla, not only having poor bone density, but also lacking adequate vertical height as a result of sinus pneumatization. The purpose of the present study was a radiologic, histological, and histomorphometrical evaluation, in humans, of specimens retrieved from sinuses augmented with decellularized bovine compact particles, after a healing period of 6 months.* Methods*. Four patients, with atrophic resorbed maxillas, underwent a sinus lift augmentation with decellularized bovine compact bone from bovine femur. The size of the particles used was 0.25–1 mm. A total of four grafts and 5 biopsies were retrieved and processed to obtain thin ground sections with the Precise 1 Automated System.* Results*. The mean volume after graft elevation calculated for each of the 4 patients was 2106 mm^3^ in the immediate postoperative period (5–7 days), ranging from 1408.8 to 2946.4 mm^3^. In the late postoperative period (6 months) it was 2053 mm^3^, ranging from 1339.9 to 2808.9 mm^3^. Histomorphometry showed that newly formed bone was 36 ± 1.6% and marrow spaces were 34 ± 1.6%, while the residual graft material was 35 ± 1.4%.* Conclusion*. In conclusion, based on the outcome of the present study, Re-Bone® can be used with success in sinus augmentation procedures and 6 months are considered an adequate time for maturation before implant placement.

## 1. Introduction

The rehabilitation of the edentulous posterior maxilla with dental implants often represents a clinical challenge due to the insufficient bone volume resulting from pneumatization of the maxillary sinus and crestal bone resorption. The resultant atrophic residual ridge is one of low-density trabecular bone with a minimal cortical component [1]. The maxillary sinus lifting technique is a common surgical technique to augment bone volume in atrophic posterior maxilla [2] and healing was allowed for about 6 to 8 months before implant insertion [3]. One of the most problematic regions is the posterior maxilla, not only having poor bone density, but also lacking adequate vertical height for endosseous implants as a result of sinus pneumatization. Sinus floor augmentation can provide the necessary bone mass to place and stabilize implants essential for the initial steps towards osseointegration [4].

Different materials are used in sinus lifting, such as autogenous bone grafts [5–7], allografts [8, 9], alloplast [8–11], and xenografts [8, 12, 13].

Bovine bone particles were used with success in sinus lifting [14]. No pathological inflammatory cell infiltrate or foreign body reactions were reported with the use of anorganic bone [15, 16]. Bovine bone has been shown to be highly biocompatible with hard oral tissues in animals and man [17, 18].

The aim of the present study was a radiologic, histological, and histomorphometrical evaluation, in humans, of specimens retrieved from sinuses augmented with decellularized bovine compact particles, after a healing period of 6 months.

## 2. Materials and Methods

**Figure 1:**
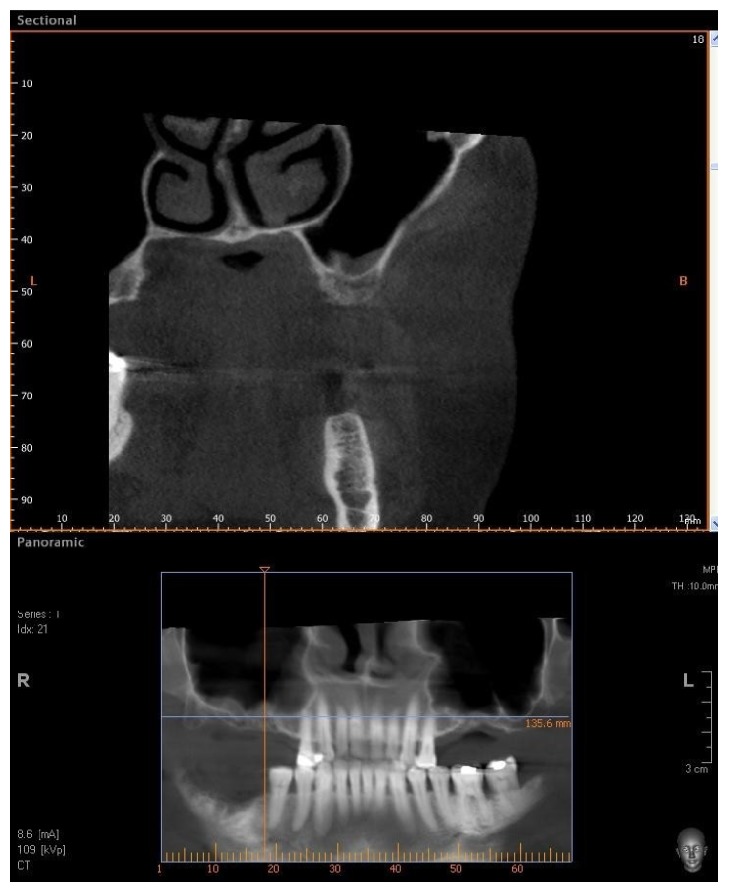
CBCT of an edentulous patient with bilateral severely atrophic maxilla.

**Figure 2:**
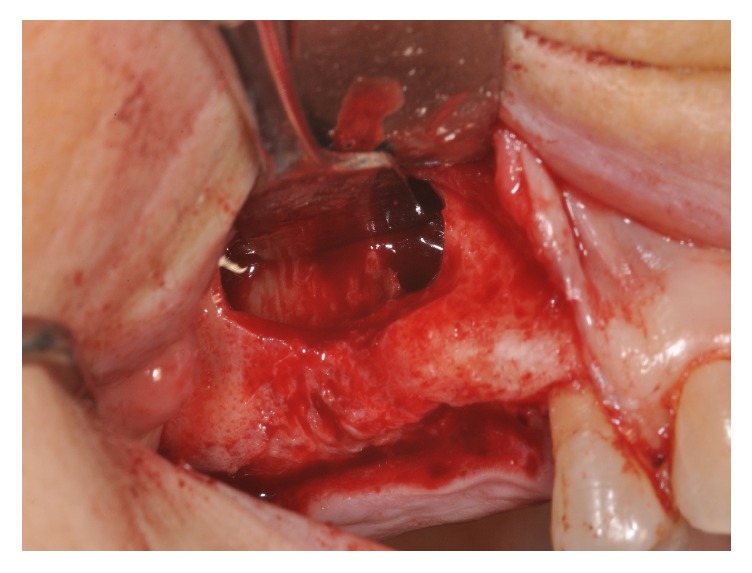
Sinus lifting procedure. The maxillary sinus lateral wall is exposed and a bone window is removed.

**Figure 3:**
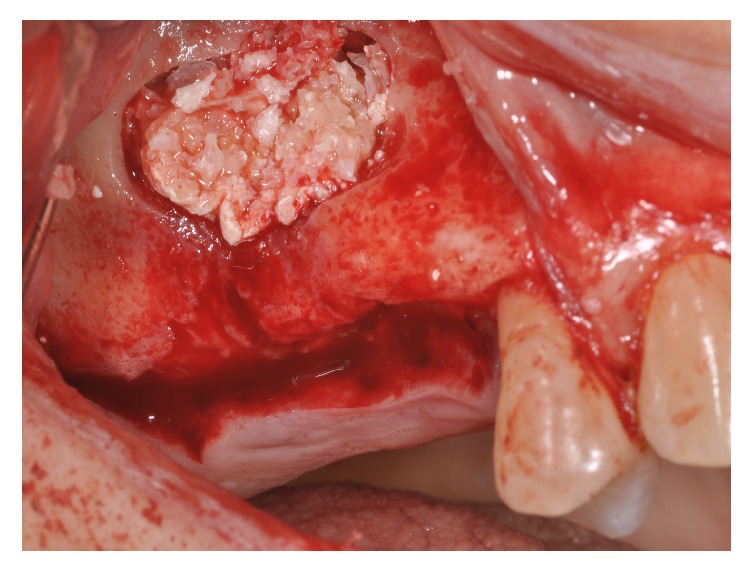
Sinus filled with cortical bovine bone.

**Figure 4:**
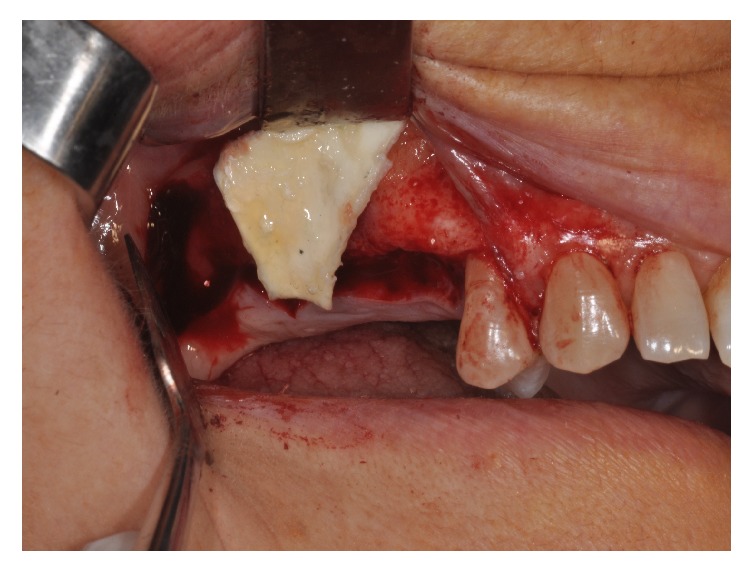
A membrane is placed over the antrostomy.

**Figure 5:**
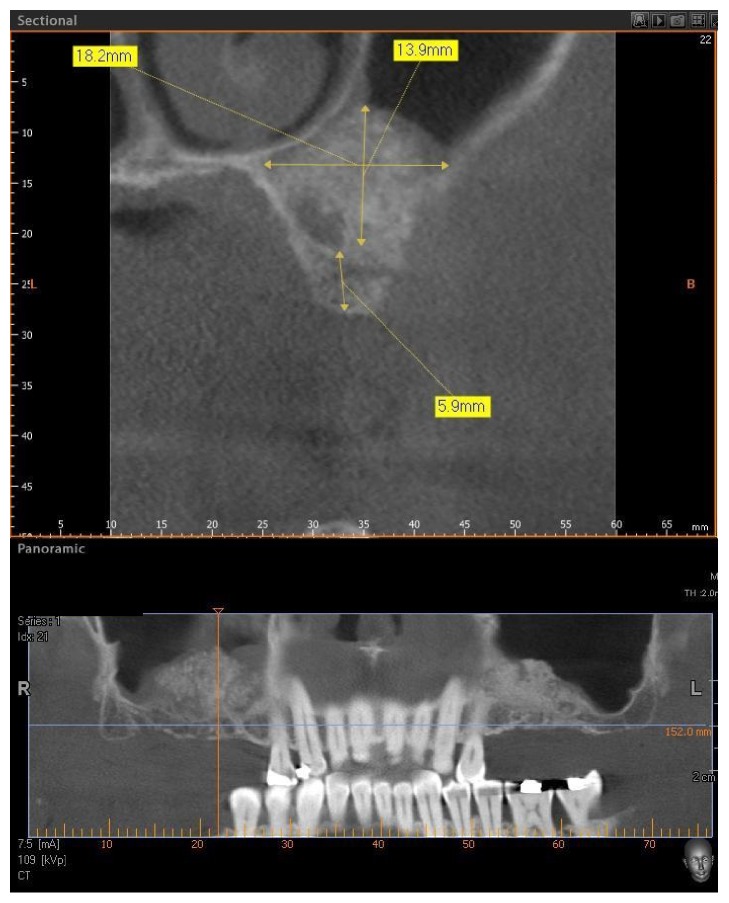
Postoperative CBCT scan panoramic view at 6 months after maxillary sinus lifting.

**Figure 6:**
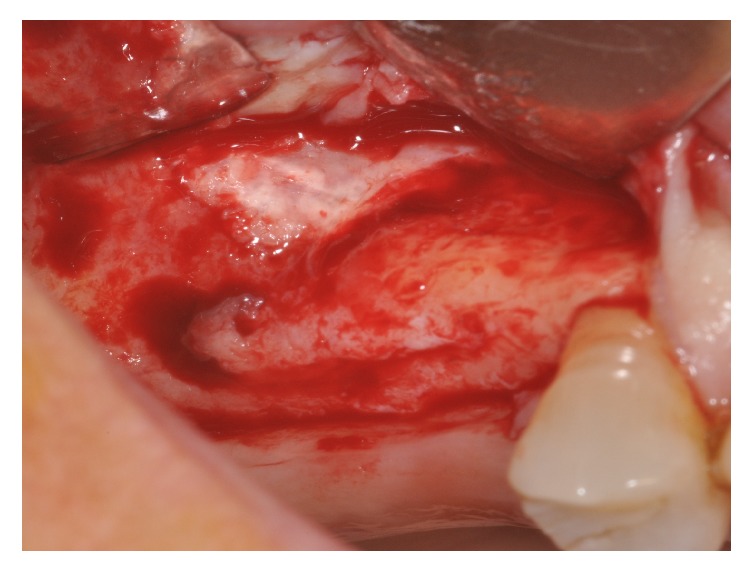
The lateral wall is completely closed by new hard tissues.

**Figure 7:**
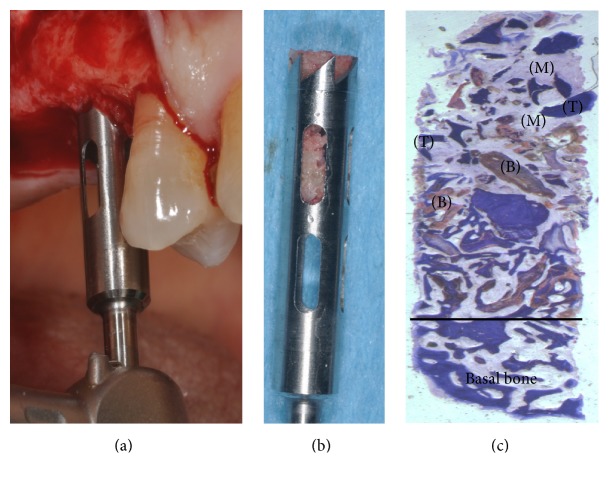
(a-b) Bone core biopsy carried out with a small trephine. (c) Newly formed trabecular bone (T) is present, with wide marrow (M) spaces and biomaterials (B). Toluidine blue 10x.

Four patients, with atrophic resorbed maxillas, underwent sinus lift augmentation with decellularized bovine compact bone from bovine femur (Re-Bone, UBGEN Padova, Italy) (Figures 1–4). The graft was condensed at each stage and a collagen membrane (SHELTER®, UBGEN Padova, Italy) The sizes of particles used were 0.25–1 mm. The sinus lift procedures were carried out as described by Boyne and James in 1980 (Figures 2–4). In all cases the sinus lifting procedure was considered to be successful and the insertion of implants of at least 12 mm was performed in all cases after 6 months. Biopsy specimens were retrieved at 6 months. A biopsy of the regenerated tissues was carried out with a small trephine under generous saline irrigation (Figures 5–7). A total of four grafts and 5 biopsies were retrieved. The cores were obtained at a mean depth of 12 mm. The specimens were retrieved, washed in saline solution, and immediately fixed in 4% paraformaldehyde and 0.1% glutaraldehyde in 0.15 M cacodylate buffer al 4°C and pH 7.4, to be processed for histology. The specimens were processed to obtain thin ground sections with the Precise 1 Automated System (Assing, Rome, Italy) [19]. The specimens were dehydrated in an ascending series of alcohol rinses and embedded in a glycolmethacrylate resin (Technovit 7200 VLC, Kulzer, Germany). After polymerization the specimens were sectioned with a high precision diamond disc at about 150 *μ*m and ground down to about 30 *μ*m. The slides were stained with basic fuchsin, toluidine blue, and von Kossa. The histochemical analysis of acid and alkaline phosphatases was carried out according to a previously described protocol. For general morphologic observations, sections were stained with toluidine blue and observed under light microscopy. To determine the relative distribution of the new matrix bone and osteoblast activity, morphological analyses were performed. A polarized light was used to distinguish lamellar bone and woven bone.

## 3. Results

The mean volume after graft elevation calculated for each of the 4 patients was 2106 mm^3^ in the immediate postoperative period (5–7 days), ranging from 1408.8 to 2946.4 mm^3^. In the late postoperative period (6 months) it was 2053 mm^3^, ranging from 1339.9 to 2808.9 mm^3^ (Figures 5 and 6).  Table 1.

No perforation of the sinus membrane was evident in any of the cases. No acute infection, with pain or fever, was observed. In all cases, bone augmentation showed hyperdensity for comparison between the immediate postoperative period and the late postoperative period, with more density than native bone at both times. The statistical analysis demonstrated a significant difference in volume alterations (*P* = 0.0119).

**Figure 8:**
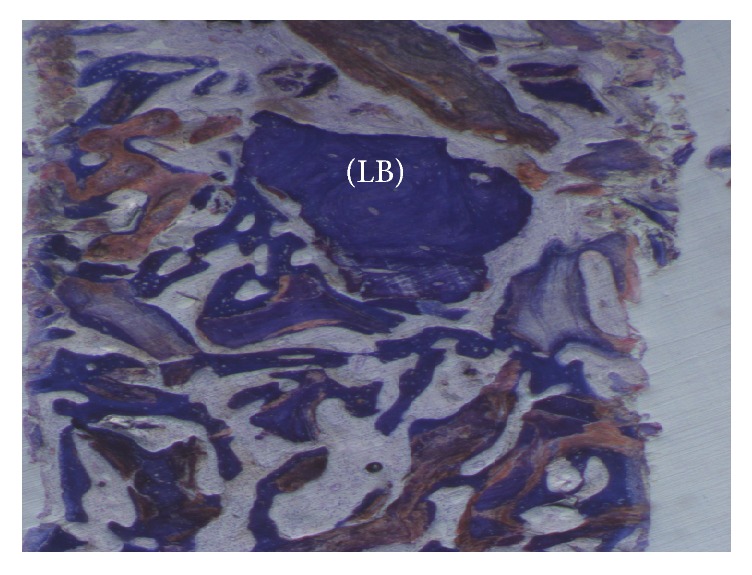
At higher magnification previous image: a few lamellar bones are visible (LB). Toluidine blue 50x.

In general, bone morphology was well present with well differentiated cellular constituents mineralized bone, osteoid, osteoblasts, osteocytes, and blood vessels. At low magnification, trabecular mature bone was observed (Figures 7 and 8). The initial formation of immature bone extending from the periphery of the bone cavities was evident. The rest of the bone cavity contained mature tissue and biomaterial with a mild inflammatory reaction.

**Figure 9:**
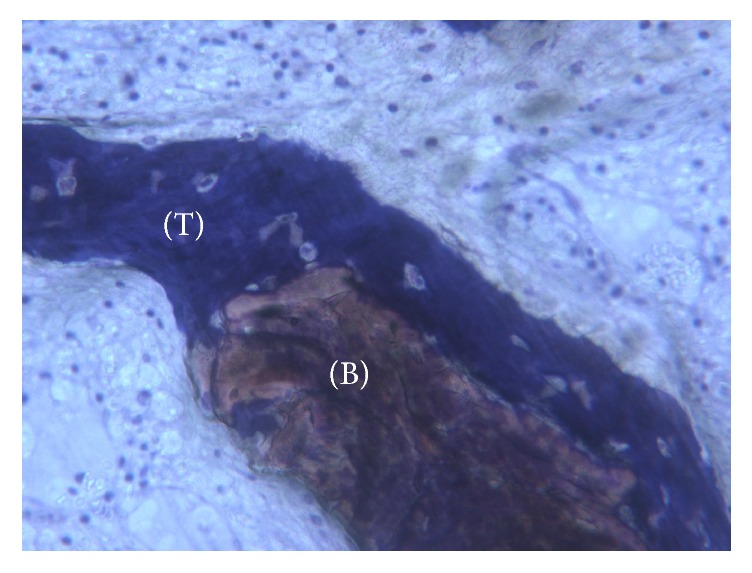
No gaps are present at the bone-particles interface, and newly formed bone is always in close contact with the particles. The biomaterial (B) seems to be totally incorporated in the trabecular bone (T). Toluidine blue 100x.

Re-Bone particles were easily distinguished from the newly formed bone: they tended to be less stained due to the low content of collagen. The particles were surrounded by newly formed bone (Figures 8 and 9). In a few marrow space areas, in which it was possible to find small capillaries, some particles were present at the interface. In some areas osteoblasts were observed in the process of posing bone directly onto the particle surface. Some positive osteoclast for acid phosphatase and a few positive osteoblast for alkaline phosphatases were observed. Histomorphometry showed that newly formed bone was 36 ± 1.6% and marrow spaces were 34 ± 1.6%, while the residual graft material was 35 ± 1.4%.

## 4. Discussion

Oral rehabilitation with osseointegrated implants is very successful and predictable in patients with normal bone volume and density, which provide adequate stabilization of implants of standard diameter and length [20]. Rehabilitation of the edentulous posterior maxilla with dental implants is often difficult because bone height is insufficient and cancellous [2].

Although there is a high risk of implant displacement/migration into the maxillary, this has been only rarely reported [10, 21]. Different biomaterials can be successfully used for sinus lifting. Many research data show that bovine bone grafting in this areas is not contraindicated and represent a procedure with low morbidity [2, 4]. This xenograft is the one most commonly used material for sinus floor augmentation and has the most powerful scientific evidence for sinus grafting [2, 4, 14, 19, 22–24] because its structure is similar to that of human [22].

In fact the outcomes of the present study showed that the Re-Bone particles appeared to be surrounded by an abundant quantity of newly formed bone. This biomaterials appeared to undergo a slow resorption process; in fact in the present study, after 6 months of observation, most of the grafting material was still in place. This study is consistent with other studies reported that the use the bovine bone as a grafting material yielded a bone formation and no presence of inflammatory cell infiltrate [25, 26]. Close contact between most of the materials and the newly formed osseous tissue was present, near but not in contact with the implant surface [14]. Several authors have discussed the use of different graft materials and have documented results both similar and varied when compared to those in the present study [14, 23]. A biomaterial similar to Re-Bone is the Bio-Oss®; this has a similar size, structure, and biological response with conducive to vessel ingrowth [15, 21]. According to our experience and previous literature, we did not observe histological differences between Bio-Oss and Re-Bone [14, 23]. The outcomes of this study revealed new bone formation around the graft particles (36 ± 1.6%) within the maxillary sinus after six months of healing. The particles showed absence of gaps at the bone-particles interface, and the bone was always in close contact with the particles. This xenograft has excellent osteoconductive properties; in fact the outcomes of the present study showed that the Re-Bone particles appeared to be surrounded by an abundant quantity of newly formed bone. Probably, also Re-Bone can be resorbed by osteoclasts [21, 24]. The grafted biomaterial was clearly distinguishable from the remaining original bone due to its density and structure. This is the first case reported in the literature to use Re-Bone granules as bone grafts in sinus lifts. The granular nature of the material facilitated its application between the sinus filling and newly formed bone.* Through* surgery, the scaffold can be easily adapted to the dimension and of the sinus. During graft placement it can quickly adsorb the blood molecules and cells promoting bone formation. Its architecture favors cell attachment and proliferation. In addition, the properties exhibited make Re-Bone a valid alternative to autogenous grafting, preventing the added morbidity of a donor surgical site. Our results were similar with a recent randomized clinical trial published in 2016 to compare histological bone quality and radiographic volume stability in maxillary sinuses grafted with porcine bone and bovine bone that confirms the validity of the bovine bone when used for sinus lifting [26]. The outcomes of the present bone core histomorphometric study showed a 35 ± 1.6% presence of Re-Bone and 36 ± 1.6% newly formed bone during the 6-month healing period. This means bone formation with low standard variation between 5 biopsies was not statistically significant. Therefore, 6 months are considered adequate time for Re-Bone maturation before implant placement or the uncovering of implants placed at the same time as grafting.

Obviously, with only 4 grafts and 5 biopsies, the data presented in this study cannot be considered conclusive. However, these results help to set practice parameters that will assure a study with a large number of patients in the future. In conclusion, the findings from the present four case reports support the use of Re-Bone as a bone substitute in maxillary sinus augmentation procedures.

## Figures and Tables

**Table 1:** Volume after graft elevation mm^3^.

N° Sinus	Immediate postoperative	After 6 months
1	1408	1339
2	2265	2265
3	1808	1800
4	2946	2808
Mean	2106,75	2053,25
SD	660	629
